# A genome size and phylogenetic survey of Mediterranean *Tripleurospermum* and *Matricaria* (Anthemideae, Asteraceae)

**DOI:** 10.1371/journal.pone.0203762

**Published:** 2018-10-09

**Authors:** Huseyin Inceer, Teresa Garnatje, Sema Hayırlıoğlu-Ayaz, Joan Pere Pascual-Díaz, Joan Vallès, Sònia Garcia

**Affiliations:** 1 Karadeniz Technical University, Faculty of Science, Department of Biology, Trabzon, Turkey; 2 Institut Botànic de Barcelona (IBB, CSIC-ICUB), Passeig del Migdia s/n, Parc de Montjuïc, Barcelona, Catalonia, Spain; 3 Laboratori de Botànica (UB)–Unitat associada al CSIC, Facultat de Farmàcia i Ciències de l’Alimentació, Universitat de Barcelona, Barcelona, Catalonia, Spain; Saint Mary's University, CANADA

## Abstract

The study of genome size variation can contribute valuable information on species relationships as well as correlate to several morphological or ecological features, among others. Here we provide an extensive report on genome sizes on genus *Tripleurospermum* and its closely related genus *Matricaria*, which are two typically Mediterranean genera particularly widespread and diverse in Turkey, the origin of most of the populations here studied. We analyse and discuss genome size variation in the first relatively complete molecular phylogenetic framework of *Tripleurospermum* (based on ITS and ETS ribosomal DNA–rDNA–regions). We find cases of intraspecific genome size variation, which could be taxonomically significant. Genome downsizing is also detected as the typical response to polyploidisation in *Tripleurospermum* taxa, being most conspicuous at the tetraploid level. Several positive correlations with genome size, including those with pollen and stomatal size or cypsela length, among others, are also found. Remarkably, taxa presenting rhizomes tend to present higher genome sizes, confirming a trend to accumulate nuclear DNA in such species, which could be explained by the nutrient reserves availability in their storage organs, allowing genome expansion, or by the lower rates of sexual reproduction in rhizomatous taxa.

## Introduction

*Tripleurospermum* Sch.Bip. is a genus of family Asteraceae, tribe Anthemideae, comprising between 30 to 40 species, depending on the authors and systematic treatments. Distributed in the Northern hemisphere, mainly in the Mediterranean basin including Europe, temperate Asia and North Africa [1, 2, 3], some taxa are also present in North America. It is particularly abundant and diverse in Turkey, its main center of diversity, where around 30 *Tripleurospermum* taxa can be found half of which endemic [4]. One of its species, *T*. *inodorum* (L.) Sch.Bip., is widespread as a weed [1]. Another Anthemideae genus with an important presence in the Mediterranean basin and closely related to *Tripleurosperum* is *Matricaria* L. The genus comprises six species [2, 5], three of them present in Turkey. Its species *M*. *chamomilla* L., a part from being widely known for its medicinal properties, is also a widespread weed [6, 7].

As in many Anthemideae and mostly due to close morphological affinity *Tripleurospermum* (subtribe Anthemidinae), *Matricaria* (subtribe Matricariinae) and, to a lesser extent, *Anthemis* L. (subtribe Anthemidinae), among others, have been confused both taxonomically and nomenclaturally [4, 8, 9, 10]. However, although some *Tripleurospermum* species were formerly assigned to *Matricaria*, later it was recognized as a different genus based on fruit features. On the one hand, the morphology of *Tripleurospermum* cypselas differ from that of those of *Matricaria* in its shape and ornamentation [2]. Besides, *Tripleurospermum* species have a tetrasporic embryo sac, a character shared with the genus *Anthemis*, while *Matricaria* and other Anthemideae present monosporic embryo sac [11, 12]. Molecular phylogenetic researches based on tribe Anthemideae are also consistent with this difference and both genera appear well separated in different and supported clades [13, 14].

Although karyology can also contribute to clarify relationships between closely related taxa [15] such as *Matricaria* and *Tripleurospermum*, little differences have been found until present regarding their chromosomes. Karyological knowledge on both genera is mostly based on chromosome counts and in some (few) karyotypes. Species of *Tripleurospermum* and *Matricaria* have the same and exclusive base chromosome number, *x* = 9, which is also the most common in tribe Anthemideae and in family Asteraceae as a whole, where it is considered the ancestral condition [16]. In certain *Tripleurospermum* species, triploid, tetraploid and pentaploid populations have been detected [17, 18], whereas in *Matricaria* only the diploid level has been recorded [19].

The study of genome size variation has shown its utility in systematic and evolutionary research in many plant groups. A first flow cytometric approach in *Tripleurospermum* was performed by our research group [17], where we measured genome sizes by flow cytometry for seven of its species and related them with features such as ploidy level, life cycle and environmental factors. Until then, only Nagl and Ehrendorfer [20] had estimated, through Feulgen cytodensitometry, the genome size for *Tripleurospermum maritimum*, the most widespread species of the genus. Very recently, Certner *et al*. [21] studied genome size variation by flow cytometry in mixed ploidy populations of *T*. *inodorum*. As for *Matricaria*, previous studies have contributed genome size information, using either Feulgen cytodensitometry [20, 22] or flow cytometry [23, 24].

The main purpose of this study is to address genome size variation in the two closely related genera *Tripleurospermum* and *Matricaria*, increasing the sample to embrace most of the species of both genera, for many of which we will provide first estimates. We will test relationships between genome size and morphological, karyological and ecological features of the species in a phylogenetic framework constructed for this purpose. This may also help clarifying systematic relationships within and between these genera and other closely related Anthemideae, as genus *Anthemis*.

## Materials and methods

### Plant materials

**Fig 1:**
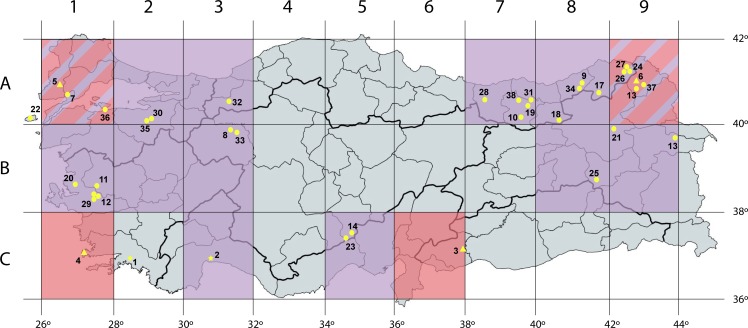
Map of Turkey with the grid system classifying the different districts as it appears in the Flora of Turkey [25]. Districts colored in violet and pink correspond to those were populations of *Tripleurospermum* and *Matricaria* were collected, respectively (both for genome size assessments and molecular phylogeny). Mixed violet and pink indicates that both *Tripleurospermum* and *Matricaria* populations were found in those districts. Codes: * *Anthemis* Δ *Matricaria* ○ *Tripleurospermum* belong to those populations used for phylogenetic reconstruction only (the corresponding number is indicated in Table 1). Map created with mapchart.net.

Cypselas of 64 populations, corresponding to 30 *Tripleurospermum* species and subspecies (42 populations), four *Matricaria* species and subspecies (21 populations) and one *Anthemis* species were collected from the wild for genome size assessments and molecular phylogenetics. We have included the species *Anthemis macrotis* (Rech.f.) Oberpr. & Vogt in the analysis since (i) it was relevant as a genus closely related to both genera and had been previously considered a member of *Matricaria* (*M*. *macrotis* Rech.f.), although a more recent taxonomic study based on molecular markers best placed it as a member of *Anthemis* [9], and (ii) no previous genome size estimate was available for the species. None of the studied species is listed as endangered or protected in any national or international legal regulation. The accessions are listed in Table 1 following the taxonomic names in the Flora of Turkey the East Aegean Islands [25], Inceer and Hayırlıoğlu-Ayaz [4] and Inceer [26]. The map in Fig 1 indicates the districts of provenance of the *Tripleurospermum*, *Matricaria* and *Anthemis* populations here studied. Specimen vouchers of the studied materials have been deposited in the herbaria of either the Karadeniz Technical University, Department of Biology (KTUB) (including the H. Inceer collection), the Centre de Documentació de Biodiversitat Vegetal, Universitat de Barcelona (BCN) and the Botanical Institute of Barcelona (BC).

**Table 1:** Provenance and voucher number of the populations here studied, together with ploidy level, chromosome number, holoploid and monoploid genome size and internal standard used. All genome sizes are first estimates in the taxa concerned with the exception of *M*. *chamomilla* var. *chamomilla*, *T*. *callosum*, *T*. *elongatum*, *T*. *maritimum*, *T*. *melanolepis*, *T*. *oreades* var. *oreades*, T. *oreades* var. *tchihatchewii*, *T*. *repens* and *T*. *sevanense*. (1) Information of locality and collection date.

Taxon	Locality^1^	Voucher	PL^2^	2n^3^	2C (SD)^4^	1Cx^5^	1C (Mbp)^6^	Internal standard
*Anthemis macrotis* (Rech.f.) Oberprieler & Vogt * (E)	C2 Muğla, Turkey: Near Köyceğiz, roadsides, 10 m a.s.l., 13.iv.2009 (1)	Inceer 711	2x	18	5.55 (0.19)	2.78	2713.95	*Petunia hybrida*
*A*. *chia* L.*	C3 Antalya, Turkey: Aksu, at the Perge ruins. 27.iii.2010 (2)	BCN 70405	2x	18**	7.4 (0.15)**	3.70**	3618.6**	Petunia hybrida**
*Matricaria aurea* (Loefl.) Sch.Bip.*	C6 Gaziantep/Şanlıurfa, Turkey; Between Nizip and Birecik, Dutlu mevkii, roadsides, near cultivated area, 440 m a.s.l., 08.v.2007 (3)	Inceer 322	2x	18***	5.04 (0.07)	2.52	2464.56	*Petunia hybrida*
*M*. *aurea*	C6 Gaziantep/Şanlıurfa, Turkey: Between Nizip and Birecik, roadsides, near cultivated area, 500 m a.s.l., 08.v.2007	Inceer 323	2x	18	4.91 (0.08)	2.46	2400.99	*Petunia hybrida*
*M*. *chamomilla* L. var. *chamomilla*	A1(E) Çanakkale, Turkey: Koru Dağı, near *Pinus brutia* forest, 70 m a.s.l. 11.v.2007	Inceer 332	2x	18	5.13 (0.07)	2.57	2508.57	*Petunia hybrida*
*M*. *chamomilla* var. *chamomilla*	C1 Muğla, Turkey: Bodrum, Ortakent, roadsides, 50 m a.s.l., 16.iv.2007	Inceer 279	2x	18	5.17 (0.08)	2.59	2528.13	*Petunia hybrida*
*M*. *chamomilla* var. *chamomilla*	C2 Muğla, Turkey: Köyceğiz, near Dalyan, cultivated area, 23 m a.s.l., 17.iv.2007	Inceer 298	2x	18	5.17 (0.06)	2.59	2528.13	*Petunia hybrida*
*M*. *chamomilla* var. *chamomilla*	C2 Muğla, Turkey: Marmaris, between Marmaris and Köyceğiz, 20 m a.s.l., 18.iv.2007	Inceer 304	2x	18	5.33 (0.09)	2.67	2606.37	*Petunia hybrida*
*M*. *chamomilla* var. *chamomilla*	C3 Antalya, Turkey: Elmalı, Sarıkaya, roadsides, 220 m a.s.l., 19.iv.2007	Inceer 312	2x	18	5.39 (0.04)	2.70	2635.71	*Petunia hybrida*
*M*. *chamomilla* var. *chamomilla*	C1 Muğla, Turkey: Bodrum, Ortakent, roadsides, 16.iv.2007, 132 m.	Inceer 283b	2x†	18†	5.30 (0.19)	2.65	2591.7	*Petunia hybrida*
*M*. *chamomilla* var. *chamomilla*	C1 Muğla, Turkey: Bodrum, Görece, roadsides, 117 m a.s.l., 16.iv.2007	Inceer 285	2x†	18†	4.96 (0.03)	2.48	2425.44	*Petunia hybrida*
*M*. *chamomilla* var. *chamomilla*	C1 Muğla, Turkey: Bodrum, Ortakent, roadsides, 89 m a.s.l., 16.iv.2007	Inceer 289	2x†	18†	5.13 (0.10)	2.57	2508.57	*Petunia hybrida*
*M*. *chamomilla* var. *chamomilla*	C3 Antalya, Turkey: Elmalı road, Yalnız village, roadsides, 450 m a.s.l., 19.iv.2007	Inceer 313	2x†	18†	5.33 (0.11)	2.67	2606.37	*Petunia hybrida*
*M*. *chamomilla* var. *chamomilla*	A1(E) Çanakkale, Turkey: Koru Dağı, near *Pinus brutia* forest, 70 m a.s.l., 11.v.2007	Inceer 332	2x†	18†	5.12 (0.02)	2.56	2503.68	*Petunia hybrida*
*M*. *chamomilla* var. chamomilla*	C1 Muğla, Turkey: Bodrum, Ortakent roadsides, 50 m a.s.l., 16.ıv.2007 (4)	Inceer 281	2x	18	5.18 (0.12)	2.59	2533.02	*Petunia hybrida*
*M*. *chamomilla* var. *recutita* (L.) Fiori	B2 İzmir, Turkey: Bozdağ, roadsides, 307 m a.s.l., 14.iv.2007	Inceer 270	2x	18	5.27 (0.04)	2.64	2577.03	*Petunia hybrida*
*M*. *chamomilla* var. *recutita*	B1 İzmir, Turkey: Yamanlar Dağı, roadsides, 750 m a.s.l., 15.iv.2007	Inceer 278	2x	18	5.18 (0.04)	2.59	2533.02	*Petunia hybrida*
*M*. *chamomilla* var. *recutita*	C1 Muğla, Turkey: Bodrum, Ortakent, roadsides, 60 m a.s.l., 16.iv.2007	Inceer 288	2x	18	5.13 (0.11)	2.57	2508.57	*Petunia hybrida*
*M*. *chamomilla* var. *recutita*	A1 Balıkesir, Turkey: Near Bandırma-Gönen road, roadsides, 150 m a.s.l., 12.v.2007	Inceer 341	2x	18	5.14 (0.10)	2.57	2513.46	*Petunia hybrida*
*M*. *chamomilla* var. *recutita*	A1 Balıkesir, Turkey: Near Bandırma-Fevzi Paşa, roadsides, 50 m a.s.l., 13.v.2007	Inceer 344	2x†	18†	5.22 (0.37)	2.61	2552.58	*Petunia hybrida*
*M*. *chamomilla* var. *recutita*	A1(E) Tekirdağ, Turkey: Near Köseilyas village, roadsides, 130 m a.s.l., 10.v.2007	Inceer 324	2x†	18†	5.24 (0.02)	2.62	2562.36	*Petunia hybrida*
*M*. *chamomilla* var. *recutita* *	A1(E) Edirne, Turkey: From Tekirdağ to Keşan, near Keşan, roadsides, 100 m a.s.l., 11.v.2007 (5)	Inceer 326	2x	18	5.13 (0.07)	2.57	2508.57	*Petunia hybrida*
*M*. *matricarioides* (Less.) Porter ex Britton*	A9 Ardahan, Turkey: Kars-Ardahan, Göle road, 1800 m a.s.l., 18.vii. (6)	Inceer 420	2x	18***	4.6 (0.07)	2.30	2249.4	*Petunia hybrida*
*Tripleurospermum baytopianum* E.Hossain (E)	A1(E) Çanakkale, Turkey: Koru Dağı, near *Pinus brutia* forest, 70 m a.s.l., 11.v.2007	Inceer 329	2x	18	4.96 (0.28)	2.48	2425.44	*Petunia hybrida*
*T*. *baytopianum* (E)	A1(E) Çanakkale, Turkey: Koru Dağı, near *Pinus brutia* forest, 70 m a.s.l., 11.v.2007	Inceer 330	2x	18	4.82 (0.10)	2.41	2356.98	*Petunia hybrida*
*T*. *baytopianum* (E)	A1(E) Çanakkale, Turkey: Koru Dağı, near *Pinus brutia* forest, 350 m a.s.l., 11.v.2007	Inceer 333	2x	18	4.65 (0.27)	2.33	2273.85	*Petunia hybrida*
*T*. *baytopianum* *(E)	A1 Çanakkale, Turkey: Koru Dağı, between Keşan and Evreşe, near *Pinus brutia* forest, 70 m a.s.l., 11.v.2007 (7)	Inceer 327	2x	18	5.02 (0.04)	1.26	2454.78	*Petunia hybrida*
*T*. *callosum* (Boiss. & Heldr.) E.Hossain*(E)	B3 Eskişehir, Turkey: Çatacık, near *Pinus* forest, roadsides,1304 m a.s.l., 27.vi.2007 (8)	Inceer 369a	4x	36	7.71 (0.14)	1.93	1885.095	*Petunia hybrida*
*T*. *caucasicum* (Willd.) Hayek *	A8 Rize, Turkey: Ayder, Kavrun, alpin meadows, 2000 m a.s.l., 11.vii.2009 (9)	Inceer 765	4x	36	8.05 (0.92)	2.01	3936.45	*Pisum sativum*
*T*. *caucasicum* *	A7 Gümüşhane, Turkey: Near Köse Dağı pass, 1852 m a.s.l., 13.vi.2009 (10)	Inceer 730	2x	18	5.16 (0.14)	2.58	2523.24	*Pisum sativum*
*T*. *conoclinum* (Boiss. & Bal.) Hayek*(E)	B2 Izmir, Turkey: Bozdağ, cultivated area, 1178 m a.s.l., 14.iv.2007 (11)	Inceer 264	2x	18	5.98 (0.11)	2.99	2924.22	*Petunia hybrida*
*T*. *conoclinum* *(E)	B2 Izmir, Turkey: Bozdağ, cultivated area, 1178 m a.s.l., 14.iv.2007 (12)	Inceer 262	2x	18	5.18 (0.06)	2.59	2533.02	*Petunia hybrida*
*T*. *corymbosum* E.Hossain*(E)	B9 Ağrı, Turkey: Suluçem, 1791 m a.s.l., 30.vi.2009 (13)	Inceer 757	2x†	18†	5.29 (0.02)	1.32	1293.405	*Petunia hybrida*
*T*. *decipiens* (Fisch. & C.A.Mey.) Bornm.*	C5 Niğde, Turkey: Ulukışla, Bolkar mountains, near mine, 1650 m a.s.l., 14.vii.2007 (14)	Inceer 395	4x†	36†	8.18 (0.11)	4.09	4000.02	*Petunia hybrida*
*T*. *disciforme* (C.A.Mey.) Sch.Bip.*	B1 Manisa, Turkey: Between Manisa and Izmir, roadsides, 1021 m a.s.l., 06.vii.2008 (15)	Inceer 592	2x	18***	4.93 (0.10)	2.47	2410.77	*Petunia hybrida*
*T*. *elongatum* (DC.) Bornm.*	A9 Ardahan, Turkey: Between Ardahan and Göle, roadsides, 1800 m a.s.l., 18.vii.2007 (16)	Inceer 423	2x	18***	4.68 (0.06)	2.34	2288.52	*Petunia hybrida*
*T*. *fissurale* (Sosn.) E.Hossain*(E)	A8 Artvin, Turkey: Yusufeli, between Yusufeli and İspir, roadsides, rocky slopes, 617 m a.s.l., 04.vi.2007 (17)	Inceer 351	2x	18***	5.33 (0.18)	2.67	2606.37	*Petunia hybrida*
*T*. *heterolepis* (Freyn. & Sint.) Bornm.* (E)	A8 Bayburt, Turkey: Kop Dağı, roadsides, damp alpine meadows, 2494 m a.s.l., 05.viii.2007 (18)	Inceer 467	4x	36	8.42 (0.26)	2.11	2058.69	*Petunia hybrida*
*T*. heterolepis*(E)	A7 Gümüşhane, Turkey: Keçikale Village, roadsides, 1618 m a.s.l., 04.vii.2007 (19)	Inceer 382b	4x	36***	8.21 (0.18)	2.05	2007.345	*Petunia hybrida*
*T*. *hygrophilum* (Bornm.) Bornm. (E)	B1 Izmir, Turkey: Yamanlar Dağı, near *Pinus* forest, open places, 887 m a.s.l., 15.iv.2007	Inceer 274	2x	18	4.94 (0.07)	2.47	2415.66	*Petunia hybrida*
*T*. *hygrophilum* (E)	B1 Izmir, Turkey: Yamanlar Dağı, roadsides, 730 m a.s.l., 15.iv.2007	Inceer 277	2x	18	4.96 (0.02)	2.48	2425.44	*Petunia hybrida*
*T*. *hygrophilum* * (E)	B1 Izmir, Turkey: Yamanlar Dağı, above Karagöl, meadows, 820 m a.s.l., 14.iv.2007 (20)	Inceer 271	2x	18	4.95 (0.05)	2.48	2420.55	*Petunia hybrida*
*T*. *inodorum* (L.) Sch.Bip.*	A9 Erzurum, Turkey: Pasinler, cultivated area, 1635 m a.s.l., 29.vi.2009 (21)	Inceer 754	4x	36	8.61 (0.15)	2.15	2105.145	*Petunia hybrida*
*T*. *inodorum* *	Münster, Germany: Wolbeck Berler Kamp. 1.vi.2012	BCN 75281	4x	36	9.32 (0.18)	2.33	2278.74	*Petunia hybrida*
*T*. *insularum* Inceer & Hayırlıoglu.-Ayaz*(E)	A1 (E) Çanakkale, Turkey: Gökçeada, 30 m a.s.l., 24.iv.2010 (22)	Inceer 789	2x	18	5.68 (0.12)	2.84	2777.52	*Pisum sativum*
*T*. *kotschyi* (Boiss.) E.Hossain *(E)	C5 Niğde, Turkey: Ulukışla, Bolkar mountains, near Karagöl, 2600 m a.s.l., 29.vii.2008 (23)	Inceer 702	4x	36***	8.29 (0.14)	2.07	2026.905	*Petunia hybrida*
*T*. *maritimum* (L.) W.D.J.Koch *	Barcelona Spain: near plaça Cerdà, Spain. 9 m a.s.l., 09.v.2005	BC 906990	2x†	18†	5.28 (0.10)	2.64	2581.92	*Petunia hybrida*
*T*. *melanolepis* (Boiss. & Buhse) Pobed.*	A9 Artvin, Turkey: Şavşat, near Çamlıbel passs, 2550–2600 m a.s.l., 20.06.2009 (24)	Inceer 741	2x	18	4.88 (0.09)	2.44	2386.32	*Petunia hybrida*
*T*. *microcephalum* (Boiss.) Bornm.*	B8 Muş, Turkey: Fallow fields, banks, roadsides, 1323 m a.s.l., 09.vii.2008 (25)	Inceer 594	2x	18***	5.49 (0.07)	2.75	2684.61	*Petunia hybrida*
*T*. *monticolum* (Boiss. & A.Huet) Bornm.*(E)	A9 Artvin, Turkey: Şavşat, alpine meadows, 2185 m a.s.l., 17.vii.2007 (26)	Inceer 416	4x	36	9.65 (0.02)	2.41	2359.425	*Petunia hybrida*
*T*. *oreades* (Boiss.) Rech.f. var. *oreades*	A8 Rize, Turkey: Anzer, roadsides,1370 m a.s.l., 19.viii.2007	Inceer 469	4x	36	8.36 (0.13)	2.09	2044.02	*Petunia hybrida*
*T*. *oreades* (Boiss.) Rech.f. var. *tchihatchewii* (Boiss.) E.Hossain*	A9 Artvin, Turkey: Şavşat, alpine meadows, 2185 m a.s.l., 17.vii.2007 (27)	Inceer 414	4x	36	9.62 (0.15)	2.41	2352.09	*Petunia hybrida*
*T*. *oreades* *	A7 Giresun, Turkey: Kümbet, near Şehitler pass, roadsides, meadows, 1719 m a.s.l., 21.vii.2008 (28)	Inceer 658	4x	36	8.9 (0.17)	2.23	2176.05	*Petunia hybrida*
*T*. *parviflorum* (Willd.) Pobed.	C3 Antalya, Turkey: Korkuteli, 965 m a.s.l., 19.iv.2007	Inceer 315	2x	18	6.01 (0.07)	3.01	2938.89	*Petunia hybrida*
*T*. parviflorum*	B2 Izmir, Turkey: Bozdağ, roadsides, 1154 m a.s.l., 14.iv.2007 (29)	Inceer 266	2x	18	6.15 (0.04)	3.08	3007.35	*Petunia hybrida*
*T*. *pichleri* (Boiss.) Bornm.* (E)	A2 Bursa, Turkey: Uludağ, meadows, damp woods, near hotels, 1828 m a.s.l., 11.vi.2008 (30)	Inceer 553	4x	36***	8.56 (0.20)	2.14	2092.92	*Petunia hybrida*
*T*. *repens* (Freyn & Sint.) Bornm.* (E)	A7 Gümüşhane, Turkey: Gezge Village, meadows, 1987 m a.s.l., 08.vii.2007 (31)	Inceer 385	4x	36	8.56 (0.16)	2.14	2092.92	*Petunia hybrida*
*T*. *rosellum* (Boiss. & Orph.) Hayek var. *album* E.Hossain* (E)	A3 Bolu, Turkey: Near Abant Lake, meadows, 1331 m a.s.l., 12.vi.2008 (32)	Inceer 555	2x	18***	4.65 (0.07)	2.33	2273.85	*Petunia hybrida*
*T*. *sevanense* (Manden.) Pobed.*	B3 Eskişehir, Turkey: Çatacık, near *Pinus* forest, roadsides, 1304 m a.s.l., 27.vi.2007 (33)	Inceer 369b	4x	36	8.40 (0.13)	2.10	2053.8	*Petunia hybrida*
*T*. *subnivale* Pobed.*	A8 Rize, Turkey: Ayder, Kavrun, alpine meadows, 2278 m a.s.l., 23.vii.2008 (34)	Inceer 672b	5x	42–48	13.11 (0.33)	2.62	2564.316	*Petunia hybrida*
*T*. *tempskyanum* (Freyn & Sint.) Hayek*(SE)	A2 Bursa, Turkey: Uludağ, near hotels, meadows, open places, 1815 m a.s.l., 25.vi.2009 (35)	Inceer 751	4x	36	8.94 (0.10)	2.24	2185.83	*Petunia hybrida*
*T*. *tenuifolium* (Kit.) Freyn*	A1 Balıkesir, Turkey: Erdek, Kapu Dağı, roadsides, 437 m a.s.l., 16.v.2009 (36)	Inceer 722	4x	36	9.11 (0.08)	2.28	2227.395	*Petunia hybrida*
*T*. *transcaucasicum* (Manden.) Pobed.	A9 Ardahan, Turkey: Between Göle and Kars, near Balçeşme, 2115 m a.s.l., 18.vii.2007	Inceer 429	2x	18	4.98 (0.08)	2.49	2435.22	*Petunia hybrida*
*T*. *transcaucasicum* *	A9 Ardahan, Turkey: Between Ardahan and Göle, roadsides, 2115 m a.s.l., 18.vii.2007 (37)	Inceer 427	2x	18	5.16 (0.07)	2.58	2523.24	*Petunia hybrida*
*T*. *ziganense* Inceer & Hayırlıoğlu.-Ayaz*(E)	A7 Gümüşhane, Turkey: Zigana Dağı, between Zigana pass and Torul, 1300 m a.s.l., 02.vi.2009 (38)	Inceer 723	2x	18	4.82 (0.09)	2.41	2356.98	*Petunia hybrida*

(1) Numbers in brackets correspond to the location of each population in the map of Fig 1, and codes at the beginning correspond to the grid system classifying the different districts as it appears in the Flora of Turkey [25].

(2) Ploidy level.

(3) Somatic chromosome number.

(4) Holoploid genome size and standard deviation in brackets.

(5) Monoploid genome size.

(6) Genome size in Mbp. (E) endemic to Turkey. (SE) subendemic toGreece and Turkey.

(*) Taxa used for molecular phylogenetic analyses.

(**) Data from [56].

(***) Data from [18].

(†) Ploidy level and chromosome number inferred from genome size data.

### Molecular techniques: DNA extraction, amplification and sequencing

Total genomic DNA was extracted using either the CTAB method [27] as modified by [28] or the Nucleospin Plant (Macherey-Nagel, GmbH et Co., Düren, Germany), depending on the quality of the vegetal material. Polymerase chain reaction (PCR) was performed by using an MJ Research Inc. thermal cycler (Watertown, Massachusetts, USA) in a 25 μL volume. Direct sequencing of the amplified DNA segment was performed with the Big Dye Terminator Cycle Sequencing v3.1 (PE Biosystems, Foster City, California, USA). Nucleotide sequencing was carried out at the Serveis Científics i Tecnològics (Universitat de Barcelona) on an ABI PRISM 3700 DNA analyzer (PE Biosystems, Foster City, California, USA). *ITS region*—Double-stranded DNA of the ITS region (including ITS1, 5.8S gene, and ITS2) was amplified by PCR with ITS1f and ITS4r primers [29]. The PCR profile used for amplification was 94°C 3min; 30 × (94°C 20 s; 55°C 1 min; 72°C 1 min); 72°C 10 min. Only the ITS4 primer was used for sequencing in most cases. *ETS region*—Double-stranded DNA of the ETS region was amplified with the ETS1f and 18SETSr primers [30]. The PCR profile used for amplification was 94°C 3min; 30 × (94°C 20 s; 50°C 1 min s; 72°C 1 min); 72°C 10 min. Both ETS1f and 18SETS were used as sequencing primers, and also the internal primers AST1F and AST1R [31] were used occasionally.

### Flow cytometric measurements

For flow cytometric measurements of leaf tissue were obtained from seeds grown in pots in the greenhouse of the Faculty of Pharmacy and Food Sciences, Universitat de Barcelona. Five individuals per population of each species were studied, and two samples of each were individually processed. *Petunia hybrida* Vilm. ‘PxPc6’ (2C  =  2.85 pg) and *Pisum sativum* L. ‘Express Long’ (2C  =  8.37 pg) were used as the internal standards [32]. Fresh leaf tissue for the standard and the target species were chopped together in 600 μl of LB01 buffer (8% Triton X-100) [33] supplemented with 100 μg/ml ribonuclease A (RNase A, Boehringer, Meylan, France) and stained with 36 μl of 1 mg/ml propidium iodide (Sigma-Aldrich, Alcobendas, Madrid) to a final concentration of 60 μg/ml, and kept on ice for 20 min. The fluorescence measurements were performed using an Epics XL flow cytometer (Coulter Corporation, Miami, FL, USA) at the Centres Científics i Tecnològics, University of Barcelona. More details about the method are in [34]. The data were submitted to the Genome Size in Asteraceae Database (GSAD) [35].

### Phylogenetic analyses and reconstruction of character evolution

The nuclear ribosomal DNA dataset (1075 concatenated bp) includes ITS (excluding the 5.8S) and 5’ ETS sequences (636 and 439 bp, respectively) for 45 taxa out of which 34 belong to *Tripleurospermum*, four to *Matricaria* and the remaining seven to different species of genera *Achillea* (GenBank accession number–hereafter GB–: AY603251), *Anacyclus* L. (GB: AY603258, GU818490, GU818112) and *Tanacetum* L. (GB: EF577323, AB359894), known to occupy an intermediate position between *Tripleurospermum* and *Matricaria* in several phylogenetic treatments of tribe Anthemideae [2] and in [36]. One species of *Artemisia* was used as outgroup (GB: HQ019060, HQ019018). The newly sequenced regions of species from *Tripleurospermum* (34 taxa), *Matricaria* (four taxa) and *Anthemis* (two taxa) are deposited in GenBank under the accession numbers MG740672-MG740711 (ITS) and MG725262-MG725301 (ETS) (release date 1^st^ January 2019 if not published before).

The two sequence matrices obtained with the nuclear molecular markers were manually edited and concatenated (S1 File) with BioEdit v. 7.1.3.0 [37] and MAFFT [38]. 54 gaps or indel characters were coded with FastGap [39] as 0/1 (absence/presence) and added to the data matrix as a separate partition.

The phylogenetic analyses were performed in the CIPRES Science Gateway [40]. Bayesian Inference phylogenetic analysis was performed in MrBayes v.3.2.6 [41] using the GTR+I+G model previously determined from jModeltest v.2.1.6 [42] under the Akaike information criterion [43]. The Markov chain Monte Carlo (MCMC) sampling approach was used to calculate posterior probabilities (PP). Four consecutive MCMC computations were run for 100,000,000 generations, with tree sampling every 10,000 generations. The first 25% of tree samples were discarded as the burn-in period. PP were estimated through the construction of a 50% majority rule consensus tree.

### Statistical analyses

All data manipulations and statistical analyses were performed with RStudio, v.0.98.1078, a user interface for R [44]. The phylogenetic generalised least squares (PGLS) algorithm as implemented in the *nlme* package for R (Version 3.1–118) was used to analyse variation of genome size with respect to karyological (chromosome number, ploidy level) and morphological traits (pollen polar axis and equatorial diameter, size of the stomata, length and width of the cypsela, size of the plant, capitulum type (homogamous, heterogamous), presence of mucilage in cypsela, presence of rhizome), as well as other features such as life cycle, altitude, habitat and invasive behavior, following Olanj *et al*. [45]. The information used for the analyses is presented in S1 Table, together with genome sizes of the accompanying species of other genera, which have been obtained from the GSAD database [35]. Additionally, regular statistical analyses of regression, one-way analysis of variance (ANOVA) and Shapiro–Wilk test for normality were performed, without considering phylogenetic relationships between taxa. The packages *ape* and *geiger* were also required for the phylogenetic-statistical analyses, as well as the package *agricolae* for LSD tests. Since in most cases datasets were not normally distributed, we also performed non-parametric tests such as Spearman rank correlation, the Kruskal-Wallis test by ranks and multiple comparison tests after Kruskal-Wallis (using the *pgirmess* package).

## Results

### Phylogenetic relationships between *Tripleurospermum* and *Matricaria*

**Fig 2:**
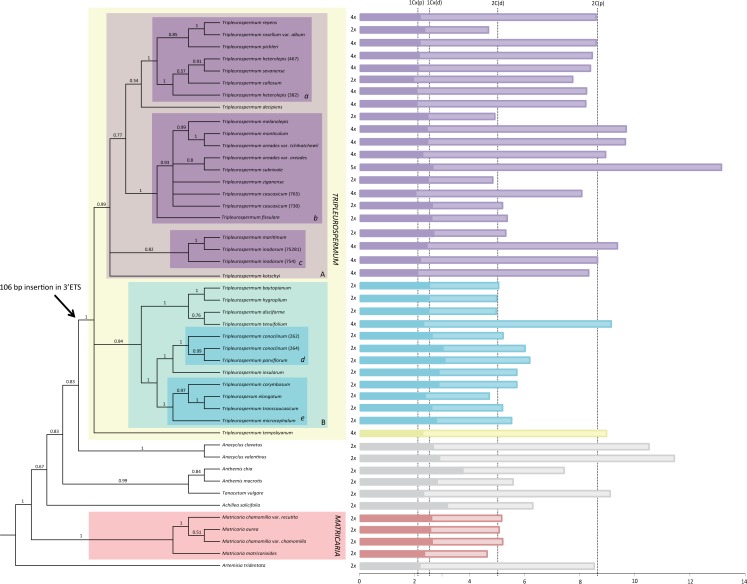
Molecular phylogenetic reconstruction obtained through combined analysis of ITS and 3’ETS sequence data for 45 taxa. The majority rule consensus tree (50%), based on Bayesian MCMC inference and with Bayesian clade-credibility values (posterior probability > 0.5) above branches, is displayed. A representation of genome sizes in bars (2C and 1Cx, in darker colours) is displayed in the right part of the image. Note, average higher genome sizes of species from clade A (purple) as compared to species from clade B (blue) in *Tripleurospermum*.

Here we contribute a preliminary molecular phylogenetic framework for *Tripleurospermum* and related genera, in which to analyse genome size variation together with other traits of the species. Genus *Tripleurospermum* appears as monophyletic and strongly supported (PP = 1.00) in our phylogenetic reconstruction (Fig 2). All *Tripleurospermum* taxa possess a 106 bp insertion in the 5’ ETS which clearly defines the group. Relationships within the genus point to two large groups highly supported (PP = 0.99 and 0.94), and several other clades at lower hierarchical levels also show strong statistical support (PP = 1.00). Although the support is moderate (PP = 0.83) the sister taxa for *Tripleurospermum* could be species from genus *Anacyclus*. Species from genera *Anthemis*, *Tanacetum* and *Achillea* occupy intermediate positions between *Tripleurospermum* and *Matricaria*. The latter genus also appears as monophyletic and strongly supported (PP = 1.00). Both genera, *Tripleurospermum* and *Matricaria*, are clearly independent in this phylogenetic framework, although both grouped with the remaining genera in a highly supported clade whose outgroup is *Artemisia*.

### Genome size variation

The present study expands genome size knowledge in *Tripleurospermum* up to 83% of the accepted taxa (25 out of 30 taxa according to the Euro+Med PlantBase [46]). There are also some species, such as *T*. *melanolepis* (Boiss. & Buhse) Pobed. and *T*. *transcaucasicum* (Manden.) Pobed., that do not appear in this database but are included as accepted names in The Plant List ([47], accepting 38 species in the genus), for which we also contribute new genome sizes. Our contribution to *Matricaria* expands to 50% the genome size knowledge on the genus, while for *Anthemis* it is much smaller, since we only add one species to the about 150 accepted species. The basic chromosome number of all taxa (*Tripleurospermum*, *Matricaria* and *Anthemis*) is *x* = 9 and ploidy level of the 64 populations here studied ranges from diploid (46 populations, 71.9%), to pentaploid (albeit only one population of *T*. *subnivale*), being the remaining 17 populations (26.6%) tetraploid.

Table 1 provides holoploid genome sizes (2C), ranging from 4.65 to 13.11 pg in *Tripleurospermum* and from 4.6 to 5.39 pg in *Matricaria*. The average half peak coefficient of variation (HPCV) corresponding to 10 samples of five individuals was 4.5% for the problem plant and 3.2% for the standards, indicating the good quality of the flow cytometric assessments. We found low intraspecific genome size differences in several cases in which two or more populations had been assessed in this study: 0.4% between three populations of *T*. *hygrophilum* (Bornm.) Bornm., 2.34% between two populations of *T*. *parviflorum* (Willd.) Pobed., 2.55% between two populations of *T*. *heterolepis* (Freyn. & Sint.) Bornm. and 2.64% between two populations of *Matricaria aurea*. Moderate intraspecific differences were detected between between 11 populations of *Matricaria chamomilla* (8.66%) and seven populations of *M*. *chamomilla* var. *recutita* (L.) Fiori (5.18%), four populations of *T*. *baytopianum* E. Hossain (7.95%) and two populations of *T*. *inodorum* (8.25%). The highest percentage difference was found between two populations of *T*. *conoclinum* (15.44%) and also between three populations and two varieties of *T*. *oreades* (15.07%). Within *Tripleurospermum*, two supported clades can be clearly distinguished (A and B in Fig 1). Ploidy levels are significantly different between these clades (Pearson’s Chi-squared test *X*^*2*^ = 18.514, *p* = 1.656·e^-05^), in which species in clade A are all tetraploid except six (out of 21), while species in clade B are all tetraploid with the exception of one (out of 12). This is also reflected as significantly different holoploid genome sizes between both clades (*p* = 6.47·e^-05^). However, holoploid genome sizes (2C) of diploid species in both clades are not significantly different (*p* = 0.221).

Table 2 summarizes the main results of the statistical analyses performed. Using the phylogenetically generalized least squares method (PGLS) we found that holoploid genome size (2C) is significantly and positively correlated with ploidy level (*p* = 0.0000) and chromosome number (*p* = 0.0000). Monoploid genome size (1Cx) decreases significantly with both (*p* = 0.0000), which points to certain degree of genome downsizing. Other morphological characters that have produced significant relationships with genome size, considering phylogenetic relationship between taxa, are: size of the plant (positive correlation, *p* = 0.0216), presence of mucilage in cypselas (negative correlation, *p* = 0.0436) and presence of rhizome (positive correlation, *p* = 0.0034). Regarding the habitat, species living in alpine environments present significantly higher genome sizes than those living in the remaining habitats (2C = 9.23, *p* = 0.0007) while species inhabiting lower, ruderal areas, show significantly lower C-values (2C = 5.51, *p* = 0.043). As for the geographical distribution, differences are not significant (though nearly) between species with a large (2C = 6.26, *p* = 0.0568), medium (2C = 6.45, *p* = 0.063) or restricted (2C = 6.50, *p* = 0.0523) area. The other features tested against genome size (pollen polar axis and equatorial diameter, dimensions of cypsela, size of stomata, capitulum type, altitude and life cycle) did not show any phylogenetically significant correlation. However, when the correlations were performed without considering the phylogenetic relationships between taxa, pollen polar axis and equatorial diameter were significantly and positively correlated with genome size (*p* = 0.002 and *p* = 0.003, respectively), as it was the length of the cypsela (*p* < 0.0001), the size of the stomata (*p* < 0.0001) and the altitude (*p* = 0.0071).

**Table 2:** Results of the statistical analyses and comparisons, using the ordinary tests (mostly analysis of regression and ANOVA) and the phylogenetically based generalized least squares (PGLS) algorithms.

	Ordinary tests	PGLS test
**2C vs. ploidy level**	**p < 0.0001 (positive correlation)**	
**1Cx vs. ploidy level**	**p < 0.0001 (negative correlation)**	
**2C vs. pollen polar axis**	**p = 0.02 (positive correlation)**	Nonsignificant
**2C vs. pollen equatorial diameter**	**p = 0.03 (positive correlation)**	Nonsignificant
**2C vs. cypsela length**	**p < 0.0001 (positive correlation)**	Nonsignificant
**2C vs. cypsela width**	Nonsignificant	Nonsignificant
**2C vs. plant size**	Nonsignificant	**p = 0.0216** **(positive correlation)**
**2C vs. stomatal size**	**p < 0.0001 (positive correlation)**	
**2C vs. altitude**	**p = 0.00709 (positive correlation)**	Nonsignificant
**2C vs. mucilage**	**p = 0.008939**	**p = 0.0436**
	presence (2C $\bar{x}$ = 5.66)	
	absence (2C $\bar{x}$ = 7.72)	
**2C vs. habitat type**	**alpine (2C** $\bar{x}$ **= 9.74) (p = 0.00874 / p = 0,0007))**	
	**miscellaneous (2C** $\bar{x}$ **= 4.85) (nonsignificant / p = 0.0218)**	
	open places (2C $\bar{x}$ = 5.31)	
	roadsides (2C $\bar{x}$ = 6.59)	
	meadows (2C $\bar{x}$ = 6.73)	
**2C vs. rhizome**	**p = 0.000179**	**p = 0.0034**
	presence (2C $\bar{x}$ = 8.36)	
	absence (2C $\bar{x}$ = 6.35)	
**2C vs. life cycle**	Nonsignificant differences between groups	
	annual (2C $\bar{x}$ = 6.53)	
	biennial (2C $\bar{x}$ = 7.41)	
	perennial (2C $\bar{x}$ = 7.19)	
**2C vs. distribution range**	Nonsignificant differences between groups	
	large (2C $\bar{x}$ = 7.22)	
	medium (2C $\bar{x}$ = 7.05)	
	small (2C $\bar{x}$ = 6.97)	
**2C vs. capitulum type**	Nonsignificant differences between groups	
	discoid capitulum (2C $\bar{x}$ = 5.88)	
	disciform capitulum (2C $\bar{x}$ = 6.55)	
	radiate capitulum (2C $\bar{x}$ = 6.97)	

## Discussion

### Phylogenetic placement of *Tripleurospermum* and *Matricaria*, and a mention to *Anthemis macrotis*

Understanding phylogenetic relationships within tribe Anthemideae has always been problematic. As Oberprieler *et al*. [2] stated, while the circumscription of the tribe is clear [1], the subtribal classification has caused considerable difficulties through its taxonomic history. Both *Tripleurospermum* and *Matricaria* appear as monophyletic and highly supported, and independent from each other, confirming previous works on morphological [48] and molecular bases [2]. Our results allow, in this respect, confirming the placement of *Tripleurospermum* and *Matricaria* in different subtribes, as proposed previously (Anthemidinae and Matricariinae, respectively) [5]. Phylogenetic relationships above *Tripleurospermum* are overall consistent with the recent work of Vitales *et al*. [49], placing species from genus *Anacyclus* as the likely sister group of the former genus. Besides, the species *Anthemis macrotis* occupies an intermediate position between *Tripleurospermum* and *Matricaria*, confirming [9], now with an additional molecular marker (ETS), which best placed the former *Matricaria macrotis* as a member of *Anthemis* on molecular bases. The former inclusion of this “rare and enigmatic species of the East Aegean” in *Matricaria* was based on certain morphological features (absence of receptacular scales) yet others (e.g. indumentum, achene traits) pointed to its best treatment as *Anthemis*, which was later confirmed [9].

As for intraspecific structuring, despite extensive research in the genus, in particular from the morphological [50, 51], anatomical [12, 52], palynological [53], chemical [54, 55] and karyological [18, 56] points of view, little is known about phylogenetic relationships between *Tripleurospermum* species. The reconstruction here presented has been performed with the purpose of providing a phylogenetic framework in which to analyse genome size variation, although it gives, in addition, a quite resolved picture of the interspecific relationships within the genus. As indicated previously, two large and significantly supported clades are apparent (Fig 1A and 1B) and these appear to differ on ploidy levels of their species (see the different sizes of the bars representing genome sizes in both clades in Fig 1). It is also observed that clade A is the largest and most diversified, containing 19 taxa and 18 species as compared to clade B with only 13 taxa and 11 species. Polyploidy has been linked to higher diversification rates [57] and it is likely that this has been the case in *Tripleurospermum*, in which, additionally, the polyploid taxa are more widely distributed geographically than the diploid.

Several clades observed in this phylogenetic reconstruction reflect what previous work (mostly floras) had already stated for certain species. In particular, species like *T*. *repens*, *T*. *heterolepis*, *T*. *sevanense* and *T*. *callosum*, inhabiting similar areas in North East Anatolia, had been considered closely related [25, 51]. They appear in the phylogeny in a strongly supported clade (Fig 2A); similarly, *T*. *melanolepis*, *T*. *subnivale*, *T*. *caucasicum* and both varieties of *T*. *oreades* are allied in the Flora of USSR, in the Flora of Turkey and in other taxonomic treatments [8, 25]; besides, *T*. *caucasicum* and *T*. *oreades* are usually considered as synonyms [46, 47], whereas these species are not considered as synonyms by some authors [8, 25, 26] [8]. These taxa are also members of the same highly supported clade in the molecular phylogeny (Fig 2B). Species like *T*. *maritimum* and *T*. *inodorum* (Fig 2C) also form a moderately supported clade, which is consistent with the fact that they have also been considered as synonyms of each other in several occasions (indeed one of the populations was recorded as *T*. *perforatum*, considered synonym of *T*. *inodorum*; similarly, *T*. *maritimum* has sometimes been quoted as *T*. *inodorum* ssp. *maritimum*). Other consistent groupings, such as the one formed by both populations of *T*. *conoclinum* and *T*. *parviflorum*, are probably explained by the sympatric habitat of both species (the three populations inhabit in close proximity in the area of Izmir Province). Other groupings may also respond to sympatry, such as the one uniting *T*. *corymbosum*, *T*. *elongatum*, *T*. *transcaucasicum* and *T*. *microcephalum* (Fig 2D): the studied populations are found in the close areas of Agrı, Ardahan and Kars Provinces, respectively, from East Anatolia; in the Flora of Turkey [25] also a close relationship between *T*. *corymbosum* and *T*. *transcaucasicum* had previously been suggested on a morphological basis.

### Genome size variation and genome downsizing in polyploids

The present study contributes new genome size data for 22 taxa of *Tripleurospermum*, for the species *Matricaria aurea* and one variety of *M*. *chamomilla* (*M*. *chamomilla* var. *recutita*) and for the species *Anthemis macrotis*, for whose genus only five genome size estimates were previously available. Genome sizes of seven *Tripleurospermum* species and one variety (*T*. *callosum*, *T*. *elongatum*, *T*. *maritimum*, *T*. *melanolepis*, *T*. *oreades*, *T*. *oreades* var. *tchihatchewii*, *T*. *repens* and *T*. *sevanense*) have been reassessed with respect to previous results [17], which are consistent through both research works. As for *Matricaria*, the values reported for *M*. *chamomilla* var. *chamomilla* are also consistent with previous genome size estimates of diploid populations of the species [58]. Regarding *M*. *discoidea* (synonym of *M*. *matricarioides*) there is a remarkable difference (23%) with one of the populations measured previously [22] with Feulgen microdensitometry (4.6 vs 5.66 pg). Average genome sizes of *Tripleurospermum* (at diploid level) and *Matricaria* are not significantly different (5.19 and 4.98, respectively), however the average genome size of *Anthemis* species (6.47) is significantly different from both genera (*p* = 0.0057), which adds evidence to the separation of *Anthemis macrotis* (2C = 5.55 pg) from *Matricaria*.

Although the C of the term “C-value” stands for constancy of genome size within a species [59], examples accumulate which show that, many times, this is not the case [29, 60, 61]. Although generally attributed to karyotype variations (e.g., polyploidy, aneuploidy or presence of B-chromosomes) many research works have observed intraspecific genome size variation beyond chromosomal features. However, well-known methodological variation or presence of staining inhibitors in plants must always be taken into account when discussing intraspecific genome size variation. Intraspecific genome size changes were explained by Ceccarelli *et al*. [62] as a result of quantitative modulations of DNA repeats and transposable elements. Genuine intraspecific variation, even within a population, has been documented for several species [63]. As discussed earlier [45], it is difficult to set a cut-off point from which a given percentage should be considered significant. In the previous genome size study on some *Tripleurospermum* taxa we found low genome size differences between most populations [17]. Here, with a much larger sampling in terms of both taxa and populations, we detect a moderate to high degree of variation between the two varieties of *T*. *oreades*, two populations of *T*. *inodorum* and two populations of *T*. *conoclinum*. Murray [64] related intraspecific genome size variations to microevolutionary differentiations, which could be taxonomically significant. This could be the case of the two varieties of *T*. *oreades*, whose distinct phylogenetic placement (Fig 2) would also support their separation as distinct species beyond genome size, as proposed previously on the basis of morphological and karyological characters [8, 56]. The case of *T*. *inodorum* could be explained by its widespread distribution and possible introgression or hybridization with its congeners [65]. Interestingly, however, one of the populations analysed of *T*. *inodorum* was formerly given the name of *T*. *perforatum*, considered as a synonym of *T*. *inodorum*, which is indeed the valid name for the species [47]. The fact that both populations of *T*. *inodorum* appear closely related in the phylogenetic tree would support this consideration; yet, genome size of the population named as *T*. *inodorum* is 8.25% lower than that formerly named as *T*. *perforatum*. So this difference might also be interpreted in terms of microevolutionary differentiations indicating certain speciation processes, still not enough manifested morphologically. The same could have happened between the two populations analysed of *T*. *conoclinum*: remarkably, the genome size of population 264 is closer to *T*. *parviflorum* than to that of the other *T*. *conoclinum* population 262, forming a highly supported clade in the phylogeny. As for *Matricaria*, the interpopulational differences detected in this study between *M*. *chamomilla* var. *chamomilla* and *M*. *chamomilla* var. *recutita* are moderate (below 10%), despite having assessed a relatively large number of populations (11 and seven, respectively). Our results are almost 50% lower than previously reported (2C = 7.7 pg) [20] for a population of the same ploidy level. Similarly, our genome size assessment of *M*. *matricarioides* is about 23% lower than previously reported by the same authors (2C = 5.66 pg). The different techniques used in our study (flow cytometry) and in the previous (Feulgen densitometry) could partly explain the discordance between data. However, results obtained for *T*. *maritimum* in the present study (2C = 5.28 pg) are almost equal to the previous data provided by Nagl and Ehrendorfer [20] (2C = 5.25 pg) so we cannot discard genuine interpopulational differences.

Genome downsizing is a well-known and common finding in polyploid systems [66]. It is a widespread biological response to polyploidisation, which may lead to the further diploidisation of the polyploid genome. In the case of *Tripleurospermum*, there is significant genome downsizing between diploids and tetraploids, which have around 20% less than expected monoploid genome size on average. However, the only pentaploid species of our dataset apparently does not undergo downsizing, as its monoploid genome size (1Cx = 2.62) is equivalent to that of the diploids (1Cx = 2.60). Genome downsizing has been detected in genera closely related genera to *Tripleurospermum* such as *Artemisia* [67], although the highest levels of reduction in the monoploid genome were usually found at higher ploidy levels contrary to our findings in the pentaploid *T*. *subnivale*. Maybe, the likely allopolyploidisation process responsible for the appearance of the pentaploid cytotype could imply the expansion of certain genomic repeats, which would increase its size.

### Genome size and phenotypic traits

Correlations between genome size at the phenotype scale are supported by many research works pointing to a positive relationship with traits such as seed and leaf mass [68, 69, 70], pollen size [45], and stomatal size [71], among others. In this regard, we have also detected a positive correlation between pollen dimensions, stomatal size and length of the cypsela, which support the direct effect of genome size on cell size (nucleotype theory, i.e. the indirect influence of DNA in development by the physical-mechanical effects of its mass) [72]. At a higher phenotype scale, we found comparable results with the height of the plant. Knight and Beaulieu [73] showed that the strength of the correlations between genome size decreased in predictive power with increasing phenotypic scale, yet our findings argue for such correlations also at higher scales in *Tripleurospermum*. Closely related to this argument, rhizomatous *Tripleurospermum* species show significantly higher genome sizes than those without rhizomes, as pointed previously from a more limited sampling on the genus [17]. Also in the Asteraceae genus *Artemisia*, the largest genome sizes are found in plants showing vegetative multiplication [74]. Much earlier, Rees and Jones [75] had also observed larger genome sizes in species with vegetative reproduction than in those presenting sexual reproduction from genus *Lolium*. We had previously argued that the presence of rhizomes could be related with higher rates of asexual reproduction and therefore lower incidence of meiosis as a controlling mechanism of genome size expansion, which may explain higher genome sizes in species showing such vegetative organs. Besides, Veselý *et al*. [76] explained that the universal tendency of geophytes (to which rhizomatous species belong) to possess higher genome sizes than their non-geophytic relatives could be related with the nutrient reserves availability in their storage organs. Polyploidy has also been found strongly associated with vegetative reproduction than diploids [77] and this is consistent with our findings since many rhizomatous species of our sample are polyploid (though not all).

The negative correlation between the presence of slime in cypselas (a mucilaginous layer that protects the seed and promotes its germination) and genome size/ploidy level in *Tripleurospermum* is consistent with the observations of [78, 79], which did not detect any slime production in certain polyploid *Artemisia*, while most diploids showed it. It was argued that the ability to form slime may depend on ploidy level [79]. In high *Artemisia* polyploids developmental abnormalities have been observed, such as disturbances in cypsela production or germination. Differences in gene expression have also been found in potato autopolyploid series at higher ploidies [80], so it might be possible that there is some dysfunction within the expression of genes responsible for mucilage production in cypselas of polyploid taxa, possibly explaining the usual absence of slime in polyploid *Tripleurospermum* taxa.

### Genome size, environment and ecology

The genome sizes of the diploid plants studied fall within the category of small genomes (2C values between 2.8 and 7 pg), whereas those of polyploids belong to the category of intermediate genomes (2C values between 7 and 28 pg) [81], and no large and very large genomes are present in the current set. This is consistent with the absence of extremophilous taxa among those considered, in agreement with the large genome constraint hypothesis [82], postulating that plants with large genomes are rare in extreme environments.

It has been suggested [83, 84] that an increased DNA amount was an adaptation to altitude. In the same line [85, 86, 87] found a high rate of polyploid taxa in different alpine systems. The selective advantages that polyploidy may confer could explain a better colonizing ability of alpine habitats by polyploids. Although polyploidy is probably not essential in determining species adaptation to alpine environments [34], we found that alpine *Tripleurospermum* taxa are usually polyploid and present higher genome sizes. Consistently, species inhabiting ruderal areas present lower C-values and genome size is positively correlated with altitude. However, this correlation may not be true in all systems and the relationship between genome size, polyploidy and altitude is probably multifactorial. For example, Mas de Xaxars *et al*. [34] found mostly diploid species in the clade comprising high mountain *Artemisia*. Indeed, our previous work on *Tripleurospermum*’s genome size variation [17] detected a negative correlation with altitude, although the sampling was much more reduced.

Some of the taxa here studied have a wide distribution, and are even considered as invasive (e.g. the weed *T*. *maritimum*), while some others have a more restricted geographical range, being endemic to very narrow areas (e.g. *T*. *baytopianum*, *T*. *corymbosum*, *T*. *heterolepis*). Although we failed to find significant differences, species with a wider distribution tend to present smaller genome sizes than those from restricted areas. Similar findings were reported previously in other groups [67]. There is evidence that a small genome size could be related to invasiveness [69, 88] and hence a wider distribution, although the reasons of this relationship are badly understood. Perhaps, a small(er) genome size would favor faster generation times that may contribute to wider geographical distributions.

## Concluding remarks

Molecular phylogenetic data, not always in agreement with classical classifications, have proved themselves here useful to confirm the monophyly of both genera addressed, *Tripleurospermum* and *Matricaria*. This was already suggested by morphological traits, irrespective of the fact that several taxa have been the object of nomenclatural combinations under both genera. We have also shown here that the study of genome size variation within a genus, or closely related genera, can provide interesting hypotheses to understand certain morphological or ecological traits observed in plants, such as the results obtained on e.g. higher genome sizes in rhizomatous taxa or absence of slime in polyploids. Our interpretations may be backed, and may be applied in a more general scope, if similar studies in other genera raise similar conclusions, but such works are still scanty in the literature.

## Supporting information

S1 File DNA sequence matrix of ITS+ETS rDNA regions for the studied species. (FAS)

S1 Table Data used for the statistical analyses together with source references. (DOCX)
