# Supplementary material for: A genome size and phylogenetic survey of Mediterranean Tripleurospermum and Matricaria (Anthemideae, Asteraceae)
Source: PLoS One. 2018 Oct 9;13(10):e0203762. doi: 10.1371/journal.pone.0203762 (PMC6177153; doi:10.1371/journal.pone.0203762)
Supplement: S1 Table — (DOCX) [file pone.0203762.s002.docx]

|  | **Taxa** | **2C** | **2n** | **Ploidy level** | **Plant size (cm)** | **Cypsela dimensions (L x W) (mm)** | **Pollen dimensions (P x E) (µm)** | **Stomata size (µm)** | **Altitude (m)** | **Capitulum** | **Mucilage in cypsela** | **Rhizome** | **Distribution** | ***Distr. codes^1^*** | **Habitat** | ***Hab. codes^2^*** | **Life cycle** | ***Life cycle codes^3^*** |
| --- | --- | --- | --- | --- | --- | --- | --- | --- | --- | --- | --- | --- | --- | --- | --- | --- | --- | --- |
| 1 | *Achillea salicifolia* | 6.26 | 18 | 2x | - | - | - | - | - | - | - | Present | - | - | - | - | Perennial | *P* |
| 2 | *Anacyclus clavatus* | 10.48 | 18 | 2x | - | - | - | - | - | - | - | Absent | - | *L* | - | - | Annual | *A* |
| 3 | *Anacyclus valentinus* | 11.4 | 18 | 2x | - | - | - | - | - | - | - | Absent | - | *L* | - | - | Annual | *A* |
| 4 | *Anthemis chia* | 7.4 | 18 | 2x | - | - | - | - | - | - | - | - | - | *M* | - | - | - | - |
| 5 | *Anthemis macrotis* (711) | 5.55 | 18 | 2x | 23 | 1.5 x 2 | 23.9 x 26 | - | 10 | Radiate | Present | Absent | Greece, Turkey, endemic | *S* | Roadsides | *R* | Annual | *A* |
| 6 | *Artemisia tridentata* | 8.49 | 18 | 2x | - | - | - | - | - |  |  | Present | - | *L* | - | - | Perennial | *P* |
| 7 | *Matricaria aurea* (322) | 5.04 | 18 | 2x | 13 | 0.6 x 0.2 | 18.7 x 20.3 | - | 440 | Discoid | Present | Absent | Spain, N. Africa, Syria, Lebanon, Turkey, Iran, Palestine, Iraq, TransCaucasus | *L* | Roadsides, near *Pistacia*-*Olea* gardens | *R* | Annual | *A* |
| 8 | *M. chamomilla* (281) | 5.17 | 18 | 2x | 28 | 0.92 x 0.32 | 21.8 x 25 | - | 50 | Radiate | Present | Absent | Palestine, Iraq, N.W. India, Europe, Turkey | *L* | Roadsides, machia places | *R* | Annual | *A* |
| 9 | *M. chamomilla* var. *recutita* (326*)* | 5.13 | 18 | 2x | 28 | 0.79 x 0.32 | 23.9 x 25.5 | - | 100 | Radiate | Present | Absent | Canary I., Portugal, Spain, Turkey, Pakistan, Iraq, Japan, N. Asia, North America | *L* | Roadsides | *R* | Annual | *A* |
| 10 | *M. matricarioides* (420) | 4.6 | 18 | 2x | 18 | 1.15 x 0.5 | 24.8 x 27.2 | - | 1800 | Discoid | Absent | Absent | Scandinavia, Central Europe, Eastern Mediterranean Region, North America | *L* | Roadsides | *R* | Annual | *A* |
| 11 | *Tanacetum vulgare* | 9.07 | 18 | 2x | - | - | - | - | - | - | - | Present | - | *L* | - | - | Perennial | *P* |
| 12 | *Tripleurospermum baytopianum* (327) | 5.02 | 18 | 2x | 23 | 1.05 x 0.4 | 22.4 x 24.6 | 25.24 | 70 | Radiate | Present | Absent | Turkey, endemic | *S* | Roadsides, meadow, slopes, near *Pinus brutia* forest | *Mi* | Perennial (biennial) | *P* |
|  | **Taxa** | **2C** | **2n** | **Ploidy level** | **Plant size (cm)** | **Cypsela dimensions (L x W) (mm)** | **Pollen dimensions (P x E) (µm)** | **Stomata size (µm)** | **Altitude (m)** | **Capitulum** | **Mucilage in cypsela** | **Rhizome** | **Distribution** | ***Distr. codes^1^*** | **Habitat** | ***Hab. codes^2^*** | **Life cycle** | ***Life cycle codes^3^*** |
| 13 | *T. callosum* (369a) | 7.71 | 36 | 4x | 35 | 1.65 x 0.5 | 23.1 x 26.4 | - | 1304 | Radiate | Absent | Present | Turkey, endemic | *S* | Roadsides | *R* | Perennial | *P* |
| 14 | *T. caucasicum* (730) | 5.16 | 18 | 2x | - | 2.25 x 0.9 | 24.2 x 28.3 | - | 1852 | Radiate | Absent | Present | Caucasus, N. & N.W. Iran and Afghanistan, Turkey, Balkans | *M* | Roadsides, open slopes | *R* | Perennial | *P* |
| 15 | *T. caucasicum* (765) | 8.05 | 36 | 4x | 17 | 2.25 x 0.9 | 24.2 x 28.3 | - | 1975 | Radiate | Absent | Present | *Caucasus*, N. & N.W. Iran and Afghanistan, Turkey, Balkans | *M* | Damp alpine pastures | *A* | Perennial | *P* |
| 16 | *T. conoclinum* (262) | 5.18 | 18 | 2x | 30 | 1.5 x 0.9 | 23.7 x 26.1 | 28.56 | 1178 | Radiate | Present | Absent | Turkey, endemic | *S* | Meadows, pastures, cultivated fields | *Me* | Perennial (biennial) | *P* |
| 17 | *T. conoclinum* (264) | 5.98 | 18 | 2x | 30 | 1.5 x 0.9 | 23.7 x 26.1 | 28.56 | 1178 | Radiate | Present | Absent | Turkey, endemic | *S* | Meadows, pastures, cultivated fields | *Me* | Perennial (biennial) | *P* |
| 18 | *T. corymbosum* (757) | 5.29 | 27 | 3x | 45 | 1 x 0.6 | 23.2 x 24.4 | 30.64 | 1791 | Radiate | Absent | Absent | Turkey, endemic | *S* | Meadows, cultivated fields | *Me* | Perennial | *P* |
| 19 | *T. decipiens* (395) | 8.18 | 36 | 4x | 50 | 1.25 x 0.5 | 24.2 x 30.1 | - | 1650 | Disciform, Discoid | Absent | Absent | Iran, Afghanistan, Caucasus, Turkey, Greece? | *M* | Roadsides, rocky slopes | *R* | Biennial | *B* |
| 20 | *T. disciforme* (592) | 4.93 | 18 | 2x | 35 | 1.2 x 0.5 | 18.3 x 23.7 | 27.6 | 1021 | Disciform | Present | Absent | Turkey, Iran, Afghanistan, Turkestan | *M* | Roadsides (meadows) | *R* | Perennial (biennial) | *P* |
| 21 | *T. elongatum* (423) | 4.68 | 18 | 2x | 12 | 1.35 x 0.4 | 22.6 x 24.7 | 24.87 | 1800 | Radiate | Present | Absent | Turkey, Caucasus | *S* | Roadsides, open meadows, stream sides | *Mi* | Perennial (biennial) | *P* |
| 22 | *T. fissulare* (351) | 5.33 | 18 | 2x | 15 | 1.4 x 0.5 | 23.1 x 26.3 | 27.08 | 617 | Discoid | Present | Absent | Turkey, endemic | *S* | Roadsides, rocky slopes | *R* | Annual (biennial) | *A* |
| 23 | *T. heterolepis* (382b) | 8.21 | 36 | 4x | 25 | 1.35 x 0.4 | 25.6 x 27.7 | 35.92 | 1618 | Radiate | Absent | Absent | Turkey, endemic | *S* | Roadsides (meadows) | *R* | Biennial | *B* |
|  | **Taxa** | **2C** | **2n** | **Ploidy level** | **Plant size (cm)** | **Cypsela dimensions (L x W) (mm)** | **Pollen dimensions (P x E) (µm)** | **Stomata size (µm)** | **Altitude (m)** | **Capitulum** | **Mucilage in cypsela** | **Rhizome** | **Distribution** | ***Distr. codes^1^*** | **Habitat** | ***Hab. codes^2^*** | **Life cycle** | ***Life cycle codes^3^*** |
| 24 | *T. heterolepis* (467) | 8.42 | 36 | 4x | 25 | 1.35 x 0.4 | 25.6 x 27.7 | 35.92 | 2464 | Radiate | Absent | Absent | Turkey, endemic | *S* | Roadsides (meadows) | *R* | Biennial | *B* |
| 25 | *T. hygrophilum* (271) | 4.95 | 18 | 2x | 39 | 1.4 x 1.1 | 25.2 x 27.5 | 26.48 | 820 | Radiate | Present | Absent | Turkey, endemic | *S* | Open places, near *Pinus* forests | *O* | Perennial | *P* |
| 26 | *T. inodorum* (75281) | 9.32 | 36 | 4x | - | 1.4 x 0.9 | 25.9 x 30.1 | 32.72 | - | Radiate | Absent | - | Spain, Turkey, Italy, Greece, C., N. & W. Europe, Crime, S. Russia, , Turkestan | - | Roadsides | *R* | NA | *NA* |
| 27 | *T. inodorum* (754) | 8.61 | 36 | 4x | 30 | 1.4 x 0.9 | 25.9 x 30.1 | 32.72 | 1635 | Radiate | Absent | Absent | Spain, Turkey, Italy, Greece, C., N. & W. Europe, *Crimea*, S. Russia, , Turkestan | *L* | Roadsides | *R* | Annual | *A* |
| 28 | *T. insularum* (789) | 5.68 | 18 | 2x | 11 | 1.3 x 0.75 | unknown | - | 30 | Radiate | Present | Absent | Turkey, endemic | *S* | Open places, rocky slopes | *O* | Annual | *A* |
| 29 | *T. kotschyi* (702) | 8.29 | 36 | 4x | 16 | 1.75 x 0.9 | 24.5 x 26.3 | - | 2600 | Radiate | Absent | Absent | Turkey, endemic | *S* | Alpine (meadows) | *A* | Perennial (biennial) | *P* |
| 30 | *T. maritimum* (906990) | 5.28 | 18 | 2x | - |  |  | - | - | Radiate | Absent | - | Scandinavia, Atlantic Europe | *L* | - | - | Annual (biennial) | *A* |
| 31 | *T. melanolepis* (741) | 4.88 | 18 | 2x | 11 | 1.5 x 0.75 | 24.2 x 28.6 | - | 2555 | Radiate | Absent | Present | Turkey, Caucasus, N. Iraq, Iran | *M* | Roadsides, alpine, meadows, open places, | *Mi* | Perennial | *P* |
| 32 | *T. microcephalum* (594) | 5.49 | 18 | 2x | 45 | 1.25 x 0.75 | 19.6 x 22.9 | 25.28 | 1323 | Discoid | Absent | N | Turkey, Iran, N. Iraq | *M* | Roadsides | *R* | Perennial (biennial) | *P* |
| 33 | *T. monticolum* (416) | 9.65 | 36 | 4x | 26 | 2.1 x 0.9 | 24.3 x 25.6 | - | 2185 | Radiate | Absent | Present | Turkey, endemic | *S* | Alpine (meadows ) | *A* | Perennial | *P* |
| 34 | *T. oreades* var. oreades *(658)* | 8.9 | 36 | 4x | 25 | 1.75 x 0.75 | 24.3 x 26.7 | - | 1719 | Radiate | Present | Present | Turkey, Caucausia, Palestine | *M* | Roadsides (meadows) | *R* | Perennial | *P* |
| 35 | *T. oreades* var. *tchihatchewii* (414) | 9.62 | 36 | 4x | 25 | 1.75 x 0.75 | 23.9 x 26.9 | - | 2185 | Radiate | Present | Present | Turkey, Balkans | *S* | Alpine (meadows) | *A* | Perennial | *P* |
|  | **Taxa** | **2C** | **2n** | **Ploidy level** | **Plant size (cm)** | **Cypsela dimensions (L x W) (mm)** | **Pollen dimensions (P x E) (µm)** | **Stomata size (µm)** | **Altitude (m)** | **Capitulum** | **Mucilage in cypsela** | **Rhizome** | **Distribution** | ***Distr. codes^1^*** | **Habitat** | ***Hab. codes^2^*** | **Life cycle** | ***Life cycle codes^3^*** |
| 36 | *T. parviflorum* (266) | 6.15 | 18 | 2x | 20 | 1.25 x 0.55 | 25.8 x 28.2 | 29.69 | 1154 | Radiate | Present | Absent | Turkey, Iran, Syria, Transcaspia*,* Turkestan, Caucasus, S. & C. Russia | *L* | Roadsides | *R* | Annual | *A* |
| 37 | *T. pichleri* (553) | 8.56 | 36 | 4x | 28 | 1.65 x 0.75 | 27.9 x 30.3 | 33.08 | 1828 | Radiate | Absent | Present | Turkey, endemic | *S* | Meadows, damp woods | *Me* | Perennial | *P* |
| 38 | *T. repens* (385) | 8.56 | 36 | 4x | 40 | 1.75 x 0.8 | 22.6 x 23.1 | 37.66 | 1987 | Radiate | Absent | Present | Turkey, endemic | *S* | Meadows | *Me* | Perennial | *P* |
| 39 | *T. rosellum* (555) | 4.65 | 18 | 2x | 20 | 1.8 x 1 | 23 x 24.9 | 28.32 | 1331 | Radiate | Present | Absent | Turkey, endemic | *S* | Meadows | *Me* | Perennial | *P* |
| 40 | *T. sevanense* (369b) | 8.4 | 36 | 4x | 33 | 1.25 x 0.45 | 21.8 x 23.6 | 34.46 | 1304 | Radiate | Absent | Present | Turkey, Iran, Caucasus | *M* | Roadsides, near *Pinus* forest | *R* | Perennial | *P* |
| 41 | *T. subnivale* (672) | 13.11 | 45 | 5x | 25 | 2.25 x 1.05 | 28.9 x 32.7 | - | 2278 | Radiate | Absent | Present | Turkey, Caucasus | *S* | Alpine meadows | *A* | Perennial | *P* |
| 42 | *T. tempskyanum* (751) | 8.94 | 36 | 4x | 40 | 1.85 x 0.5 | 24.9 x 27.4 | 35.72 | 1815 | Discoid | Absent | Present | Greece, Turkey | *S* | Meadows (open places) | *Me* | Perennial | *P* |
| 43 | *T. tenuifolium* (722) | 9.11 | 36 | 4x | 70 | 1.75 x 1.15 | 25 x 27.1 | 33.88 | 437 | Radiate | Absent | Absent | Turkey, Balkans, Austria, Hungary, Romania | *M* | Roadsides | *R* | Perennial | *P* |
| 44 | *T. transcaucasicum* (427) | 5,16 | 18 | 2x | 35 | 1.25 x 0.65 | 26 x 23.9 | 28.92 | 2115 | Radiate | Present | Absent | Turkey, Caucasus | *S* | Roadsides (steppe areas) | *R* | Perennial | *P* |
| 45 | *T. ziganense* (723) | 4.82 | 18 | 2x | 23 | 1.95 x 1 | 22.7 x 27 | 28.25 | 1300 | Radiate | Present | Absent | Turkey, endemic | *S* | Open places, rocky slopes, roadsides | *Mi* | Biennial | *B* |
|  | **Data sources** | **-** | **a,b** | **-** | **c,d,e,**  **f,g** | **c,d,e,f,**  **g,h,i** | **j** | **a** | **a,d,e,f,**  **g,h,k** | **d,e,f,g,h,k** | **c,d,e,f,**  **g,h,l** | **d,e,f,g,h,**  **k,m,n** | **a,f,g,h,**  **m,n** | **-** | **a,c,d,e,**  **g,h,k** | **-** | **c,d,e,f,**  **g,h,k** | ***-*** |

**(1)** Distribution codes: Large (L), Medium (M), Small (S). **(2)** Habitat codes: Roadsides (R), Meadows (Me), Open places (O), Alpine (A), Miscellaneous (Mi). **(3)** Life cycle codes: Annual (A), Perennial (P), Biennial. (B). When biennial was reported with either annual or perennial, the second or third (usually more common) were selected for the analysis. Codification columns indicated in italics.

**References (data sources)**

(a) Inceer H. & Hayırlıoglu-Ayaz S. (2010). Chromosome numbers in *Tripleurospermum* Sch. Bip. (Asteraceae) and closely related genera: relationships between ploidy level and stomatal length. Pl Syst Evol 285: 149-157.

(b) Semple JC, Watanabe K. (2009). A review of chromosome numbers in the Asteraceae with hypotheses on chromosome base number evolution. In: *Systematics, Evolution and Biogeography of the Compositae* (Funk VA, Susanna A, Stuessy T, Bayer R, eds.). Vienna, International Association for Plant Taxonomy. Pp. 21-32.

(c) Unpublished data.

(d) Inceer H. (2015). Redescription of *Tripleurospermum heterolepis* (Asteraceae), endemic to Turkey. Phytotaxa 202: 214-218.

(e) Inceer H, Beyazoğlu O. (2004). Karyological studies in *Tripleurospermum* (Asteraceae, Anthemideae) from north-east Anatolia. Bot J Linn Soc. 146: 427-438.

(f) Inceer H, Hayırlıoglu-Ayaz S. (2008). *Tripleurospermum ziganaense* (Asteraceae, Anthemideae), a new species from north-east Anatolia, Turkey. Bot J Linn Soc. 158: 696-700.

(g) Inceer H, Hayırlıoğlu-Ayaz S. (2014). *Tripleurospermum insularum* (Asteraceae, Anthemideae), a new species from Turkey.  Ann Bot Fennici 51: 49-53.

(h) Enayet Hossain ABM. (1975) *Tripleurospermum* Schultz Bip. In: *Flora of Turkey and the East Aegean* *Islands* (Davis PH, ed.). Edinburgh, Edinburgh University Press, v.5. Pp. 295–311.

(i) Bal M. (2009). Anthemideae (Asteraceae) tribusunda karpolojik araştırmalar. Master thesis, Karadeniz Technical University, Trabzon, Turkey (in Turkish).

(j) Ceter T, Pinar NM, Inceer H, Hayırlıoğlu-Ayaz S, Yaprak AE. (2013). The comparative pollen morphology of genera *Matricaria* L. and *Tripleurospermum* Sch.Bip. (Asteraceae) in Turkey. Pl Syst Evol. 299: 959-977.

(k) Davis PH, Mill R, Tan K. (1988) Flora of Turkey and East Aegean Islands. Edinburgh, Edinburgh University Press, v.10.

(l) Inceer H. (2011). Achene slime content in some taxa of *Matricaria* L. (Asteraceae). Acta Bot Croat 70: 109-114.

(m) Pobedimova EG. (1995) *Tripleurospermum* Sch. Bip. In: *Flora U.S.S.R.* (Shishkin BK, Bobrov EG, eds.). Bishen Singh Mahendra Pal Singh, Dehra Dun, India and Koeltz Scientific Books, Koenigsten, Germany, v. 26. Pp. 181–213.

(n) Podlech D. (1986). *Tripleurospermum* Sch. Bip. In: *Flora Iranica* (Rechinger K, ed.). Graz: Gruck V. Verlagsanstalt, v. 158. Pp. 73-80.
